# Enhancing Emotion Regulation Skills in High-Risk Adolescents Due to the Existence of Psychopathology in the Family: Feasibility and Uncontrolled Pilot Study of a Group Intervention in a Naturalistic School Setting

**DOI:** 10.3390/ijerph21060738

**Published:** 2024-06-05

**Authors:** Christiana Theodorou, Maria Karekla, Georgia Panayiotou

**Affiliations:** 1 Department of Psychology, University of Cyprus, 2109 Nicosia, Cyprus; mkarekla@ucy.ac.cy; 2 Center for Applied Neuroscience, University of Cyprus, 1678 Nicosia, Cyprus; georgiap@ucy.ac.cy

**Keywords:** emotion regulation, internalizing, externalizing, prevention, socio-emotional skills, school-based intervention

## Abstract

Background: Emotion regulation skills form part of many interventions for youth with internalizing and externalizing difficulties. This pilot study examines a prevention program delivered at school to improve adolescents’ emotion regulation skills, focusing on those at risk for mental health problems. Methods: Adolescents 12–18 years old were referred to a six-session group program by their school counselors, based on inclusion criteria related to family sociodemographic and mental health characteristics. Group sessions took place during school hours to facilitate participation and reduce dropout. The intervention targeted emotion regulation skills, drawing from central components of different cognitive behavioral approaches. To assess clinical outcomes, participants answered questionnaires before and after the program, which covered emotion regulation strategies, addictive behaviors, and internalizing and externalizing symptoms. The acceptability of the program was also assessed. Results: Emotion regulation skills improved after the program, and there was a significant reduction in internalizing and externalizing problems. The program was evaluated as useful by participants. Counsellors reported satisfaction with the program. Conclusions: Targeted emotion regulation skills training is a potentially useful transdiagnostic intervention to prevent mental health problems in youth. Bringing the intervention to the school setting and involving counsellors in referring at-risk students can facilitate uptake and reduce dropout.

## 1. Introduction

Adolescence is a developmental stage with many psychosocial, biological, cognitive, social, and emotional changes [1,2]. Adolescents are commonly characterized as aggressive, opinionated, explorative, and experimenting—brave, emotional, eager, never saying no to peers among other descriptions. These characteristics can be considered as both positive and negative, as opportunities to learn about themselves, others, and the world [1,3].

Poor emotion regulation (ER) has been associated with internalizing and externalizing problems [4,5,6], including anxiety disorders [7,8], depression [9], and aggressive behavior [10,11,12,13]. In general, emotional dysregulation in internalizing disorders manifests as an extreme or persistent negative affect, while emotional dysregulation in externalizing problems manifests as emotional and behavioral lability [14,15]. Importantly, adaptive ER predicts well-being and reduced psychopathology [16,17,18,19]. For these reasons, helping children and adolescents develop ER skills may provide a pathway for the early prevention of mental health problems.

Adaptive ER encompasses emotional, behavioral, and cognitive processes that adjust emotional responses to situational demands and personal values and goals, and facilitate healthy psychosocial functioning [20,21]. Examples of ER strategies typically considered adaptive include reappraisal, problem solving, and acceptance, while maladaptive strategies include suppression, rumination, and avoidance [22,23,24,25].

Numerous psychotherapy protocols and interventions include components that aim to improve ER processes, which are believed to be trainable through psychosocial interventions. Evidence from multiple cognitive behavioral therapy (CBT)-based programs support this assumption, showing that the reduction in psychological symptoms via improvement was both directly or indirectly associated with ER skills [26,27,28,29]. Randomized controlled trials (RCTs) of programs directly focused on ER indicate significant reductions in anxiety [30,31] and emotional symptoms [32,33]. Some skills like anger management and cognitive problem solving [34,35,36] are promising for externalizing symptoms as well. Additional evidence comes from studies of interventions that include components on ER as part of more diverse treatment goals. These skills encompass techniques like relaxation, cognitive restructuring, and exposure, and show different degrees of effectiveness for internalizing externalizing problems [36,37,38].

On the other hand, despite their effectiveness, CBT (and other) treatments for adults and adolescents suffer from considerable dropout rates [39,40], an indication of low acceptability. This problem has partially been attributed to the demand for cognitive changes involved [41], but practical factors, including transportation and timing difficulties [40], and family demographics such as low maternal education and family adversity [42], as well as treatment credibility and the perception of the treatment as organized and effective are also reported as important factors in early termination [43]. These shortcomings suggest that improving the efficacy, credibility, and acceptability of ER-skills-based interventions is an important research target.

One approach in this direction is to draw effective practices for training ER skills, from various protocols, including first- (behavioral therapy), second- (cognitive therapy), and third- (acceptance-based therapies) wave CBT approaches, to capitalize on their complementary strengths [44]. For example, it has been argued that third-wave approaches, like Acceptance and Commitment Therapy (ACT), demonstrate reduced dropout rates compared to other protocols, perhaps due to an emphasis on values clarification that motivates clients to engage in the challenges of the treatment [40]. ACT focuses on the context of psychological experiences (thoughts, feelings, and sensations), rather than on symptoms, and stresses acceptance, as opposed to avoidance, and how one relates to emotions rather than attempting to change them [45,46,47,48,49].

Traditional Cognitive Behavioral Therapy (CBT), on the other hand, focuses on the idea that one needs to rationally monitor and change faulty interpretations of situations, and/or change one’s behavioral responses [50]. Additional CBT tools, like exposure, activity scheduling, relaxation, interpersonal skills, and behavior modification, and strategies specific to particular emotions like stress management, anger management, or fear inoculation [51] may be particularly relevant to adolescents facing adversity and may increase credibility among families, due to their intensive behavioral components [43].

Dialectical Behavioral Therapy (DBT) also stresses ER skills using techniques from both traditional CBT and mindfulness and acceptance approaches [52]. Its aim is the reduction in ineffective action tendencies (e.g., substance use, aggression, etc.) [52] as attempts to regulate emotions [53,54], and may be effective among emotionally labile adolescents, prone to substance use and disruptive behaviors. Combining the strengths of these approaches may radically increase the skills repertoire of adolescents, and their confidence to cope with life’s challenges, while reducing dropout and increasing intervention acceptability and uptake.

### Current Study

This pilot investigation assesses the viability and acceptability of a brief group intervention tailored for adolescents, specifically targeting ER skills, a critical mechanism implicated in emotional and behavioral challenges. Despite the general promise of prevailing CBT interventions addressing internalizing and externalizing issues, their effectiveness is moderately established, with variable treatment response outcomes [37,38,55], and concerning dropout rates, especially for adolescents. Barriers to treatment include logistical concerns such as transportation, time constraints, and treatment credibility, particularly among families with a lower SES status or those facing adversity. A preventative approach designed to enhance engagement by offering intervention during school hours, thus avoiding imposing on adolescents’ leisure time, removing the need for transportation and extra time commitments, and reducing the stigma associated with seeking external psychological services, holds potential appeal for participants and their families. The provision of the intervention within the school setting is expected to bolster its credibility, as will a focus on behavior modification through skills training. To facilitate engagement among youths most in need of prevention, i.e., those at elevated risk for internalizing or externalizing pathologies and substance use, school counselors, who are most closely acquainted with the needs of their students, actively participated in the referral process for the current intervention. All these reasons stress the importance of developing programs in the school setting, especially for students whose parents have mental illness [18,56,57]. However, there is so little research on effective school-based interventions. To enhance the “dose” of ER skills training, the intervention draws theoretically grounded components from diverse CBT approaches centering on ER, seeking to boost impact and uptake.

To assess feasibility and acceptability of this approach, we collected qualitative and quantitative data on participant satisfaction, dropout rates, and school counselor satisfaction. Although this is an uncontrolled pilot study, initial evidence on how ER training can decrease internalizing and externalizing problems and substance use is provided though a comparison of pre- and post-treatment measures. The content of the intervention is described in Table 1. The intervention had the name “Journey to Myself” and was created by the authors. The contents of the intervention were focused on paying conscious attention to emotion awareness, the ability to label and the acceptance of emotions, and then the ability to regulate them functionally.

In this prevention program, CBT techniques focused on the awareness of the correlation of cognitions–emotions–behaviors, and strategies for changing beliefs, thoughts, and behaviours, so adolescents would develop a more accurate appraisal of reality [50]. ACT focused on psychological flexibility, in which adolescents would experience their emotions and bodily sensations fully without trying to change, control, or avoid them [48]. Adolescents were taught to challenge unhelpful and unrealistic cognitions while also learning acceptance and other problem-solving skills. The main goal of DBT was to increase skills for self-regulation (such as emotion regulation and mindfulness), interpersonal effectiveness, distress tolerance, and balanced thinking and acting [52].

We expected that participants would show increased adaptive ER skills after the intervention and reduced internalizing and externalizing problems and substance use. We also predicted that the intervention would be feasible within the school setting, as indicated by counselor satisfaction and student participation, and would show acceptability as indicated by student satisfaction and the low dropout. To address these hypotheses, participants answered questionnaires before and after the program regarding ER skills and symptoms. Students and counselors reported on their satisfaction after the intervention.

## 2. Materials and Methods

### 2.1. Participants

**Figure A1:**
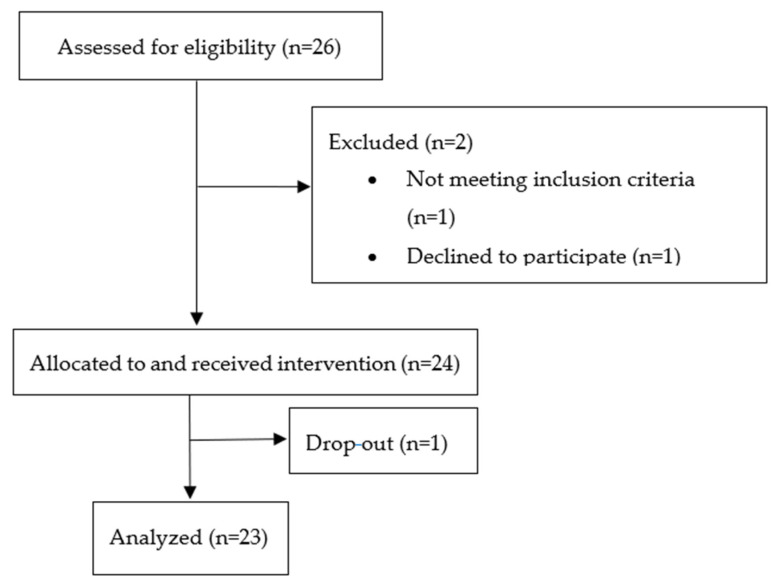
The CONSORT diagram shows the study design.

Twenty-three adolescents 12–18 years old (Μ = 15.7 years, SD = 1.94; 12 male, 11 female) participated (Figure A1), from two schools in the Nicosia district in Cyprus. All participants were assigned into four separate psychoeducation groups of 5–8 adolescents, mixed in terms of gender [58,59], taking place at their own school. Adolescents of about the same age were placed in the same group, while we avoided placing children from the same class together. School counselors nominated the adolescents who might benefit from the intervention, using inclusion criteria pertaining to family problems known to the school (addiction, psychopathology, divorce, or other psychosocial stress), or behavior problems of the adolescent. Because of the preventive nature of the program, exclusion criteria were adolescents’ existing severe psychopathology and addiction (based on questionnaire data available in school records, completed at the beginning of each school year).

According to the school records of the selected participants, among their parents, 8.7% had addiction problems, 78.3% psychopathology, and 13% faced both addiction and psychopathology. Of these parents, 47.8% were married, 39.1% divorced, 4.4% widowed, and 8.7% were single mothers, whose children had never met their father. Most parents had at least a secondary education (65.1% fathers and 56.4% mothers), 12.9% of fathers and 26.1% of mothers had tertiary education, and 8.7% of fathers and 4.3% of mothers had primary education. Regarding participants, 47.8% were living with both parents, 47.8% with one of their parents (43.5% of them with their mother and 4.3% of them with father), and 4.3% of participants were living in a state facility. Compared to the general population, where 87.23% of children live with both parents, with only 12.76% living in a single-parent home (Cyprus Statistical Service, 2011), our sample can be considered as having increased demographic risk factors. In terms of ethnicity, participants were mostly Greek-Cypriot (82.6%).

### 2.2. Measures

The following questionnaires were answered by participants before and after the intervention. Most were already translated and standardized in the Greek language. The questionnaires of emotion regulation were front- and back-translated following appropriate author approvals by the first author and a second bilingual collaborator for purposes of this study.

DERS: The Difficulties in Emotion Regulation Scale [60] is a 36-item self-report questionnaire designed to assess emotional dysregulation on six subscales: acceptance of emotions, goals, impulsivity, awareness, access to ER strategies, and emotional clarity. Each item is rated on a 1 to 5 scale (1 = almost never; 5 = almost always). Higher total scores suggest greater problems with ER. The original DERS had high internal consistency (α = 0.93) and the average internal consistency of subscales was α > 0.80 [60]. For the current sample, DERS was also very reliable (α = 0.87).

CERQ: The 36-item Cognitive Emotion Regulation Questionnaire [61] identifies cognitive emotion regulation strategies used after having experienced negative events or situations. It consists of 9 subscales; self-blame, other-blame, rumination, catastrophizing, putting into perspective, positive refocusing, positive reappraisal, acceptance, and planning. Previous studies showed that all subscales had good internal consistencies and validity (0.68 ≤ α ≥ 0.86) [61,62]. It was also highly reliable (total score, α = 0.89) in the current study.

YSR: The Youth Self-Report [63] is a widely used measure of internalizing and externalizing problems in youth. The 53 items are rated as not true (0), somewhat or sometimes true (1), and very true or often true (2). DSM-IV-based scoring assesses six diagnostic categories: affective problems (13 items), anxiety problems (6 items), somatic problems (7 items), ADHD (7 items), oppositional defiant problems (ODP), and conduct problems (CP, 15 items) [64]. The YSR has good test–retest reliability and internal consistency (α = 0.82–0.93), for the total score, the internalizing and externalizing scales (α = 0.88 and α = 0.88, respectively), and the six diagnostic categories (0.61–0.85) [65]. The Greek version showed that subscales had medium to high internal consistency (0.50 ≤ α ≥ 0.80) [64]. In the current sample, alpha for all items was 0.85 (α = 0.78 for internalizing and α = 0.87 for externalizing problems).

AUDIT: The Alcohol Use Disorders Identification Test [66] is a 10-item screening questionnaire developed by the World Health Organization to assess alcohol dependence, drinking behaviors, and alcohol-related problems. A score of >8 indicates hazardous alcohol use. The original version of the AUDIT had high internal consistency (0.75–0.94) [28,67,68]. The Greek version has high internal reliability (Cronbach α = 0.80) [69], 0.72 in an adolescents’ sample [70].

FTND: The Fagerstrom Test for Nicotine Dependence [30] assesses the intensity of nicotine addiction from cigarette smoking. It contains six items that evaluate the quantity of cigarette consumption, the compulsion to use, and dependence. Participants who score between 1 and 2 are classified as having low nicotine dependence, 3–4 as having a low to moderate dependence, 5–7 moderate dependence, and over 8 high dependence. The original version of FTND had acceptable Cronbach’s alpha reliability (alpha = 0.63). The Greek version used with adolescents had acceptable internal reliability (Cronbach’s alpha > 0.60) [71].

Acceptability: To assess the program’s acceptability, attitudes and expectations of participants for the program were measured. The intervention evaluation questionnaire developed for this purpose included four questions for participants: (1) Did you find the intervention useful? (rated as not at all, somewhat, very, and very much); (2) What did you like most about the intervention? (qualitative answer); (3) Do you want to attend a similar intervention in the future? (rated as not at all, somewhat, very, and very much); and (4) Write any comments or recommendations (qualitative answer). Dropouts were also counted.

### 2.3. Procedure

Two schools in the Nicosia District were contacted and invited to participate after the program was introduced to the school counselor. Both schools agreed to host the program. Approval for all study aspects was secured from the National Bioethics Committee and the school administration. Consent documents were signed by both parents, except when custody belonged to the Department of Social Affairs, and when it was signed by this Service and by the adolescents themselves. School counselors were asked to recommend the program to students they considered as at-risk due to family factors including psychopathology, addiction, and psychosocial risk. The counselors used information they legally had access to from the school psychologists and/or social welfare, or information provided by the student and/or parents to the school.

Specifically, the inclusion criteria were adolescents who: (a) had a family member with an addiction problem (drug or alcohol use, gambling), (b) had a family member with psychological difficulties (e.g., depression, anxiety disorder, bipolar disorder, etc.), (c) came from stressful family environments (with domestic violence, maltreatment, divorce, mourning, etc.), and (d) had good knowledge of Greek. Given the intervention’s focus on prevention, exclusion criteria included: (a) adolescents with known addiction problems themselves (daily or weekly drug use, such as cocaine, heroin, crystal meth, etc.)—alcohol and nicotine use were not considered addiction problems and adolescents would not excluded for these; (b) adolescents with severe psychopathology (e.g., diagnosed bipolar disorder, or schizophrenia); and (c) participants who were not literate in Greek.

These criteria were not disclosed to children or their families but were given to counselors to help identify adolescents who might benefit from the program. Specifically, the school counsellor informed selected adolescents whom they knew on the basis of their counseling role about the program and that it could help them improve their coping with everyday difficulties. Twenty-five of the contacted students agreed and one declined participation in the program (Figure A1). When adolescents agreed to participate, the counselor contacted parents by telephone, explained the study, and obtained written consent. Students and parents were informed that the school collaborated with the University to offer an intervention to help students learn how to manage difficult emotions. Students were also informed about confidentiality, voluntary participation, and the fact that they could discontinue at any time.

The study was conducted in accordance with the Consolidated Standards of Reporting Trials (CONSORT) guidelines for participants’ eligibility [72]. The prevention program included six sessions, delivered once a week, lasting 90 min. The program was delivered by Clinical Psychology doctoral trainees and School Psychology master-level trainees, supervised by a licensed clinical psychologist. At the end of each session, participants were given homework to practice the specific skill learnt.

## 3. Results

### 3.1. Feasibility and Acceptability

Twenty-three participants completed the study, indicating an overall 95.8% adherence rate. One adolescent dropped out after the first session. The adolescents attended, on average, 5.4 sessions (SD = 0.72) over the 6-week intervention period. Participants were generally satisfied with the intervention. More specifically, the majority of adolescents (66.7%) reported that the program was very useful and 33.3% that some of the topics were useful. When asked if they would like to participate in a similar intervention again in the future, most (62%) reported a very strong intention to do so, 23.8% some intention, and 14.2% not at all. In addition, some adolescents provided qualitative comments that showed that they enjoyed the sessions and found the practical examples very useful, but might need more of the intervention. Specifically, their comments were “I’ve received very useful and practical skills”, “I’ve had a pleasant experience being in the program”, “I am more able to manage my anger after the program”, “Now I manage anxiety better”, “I would prefer it if the program included more sessions”, and “I believe that I need more time to practice some skills”.

The two counselors at the collaborating schools were asked to comment after the conclusion of the program, but they had not attended the program. Their comments were as follows: “The program was an excellent opportunity for our students who were not able to visit a psychologist and they need to express openly their emotions”, “We could support our students by offering this program to them”, and “We noted that students improved their aggressive behavior and developed skills that made them capable to manage their outbursts after the program”. Anecdotally, several schools beyond the collaborating ones contacted the research team and requested that the program be offered at their school as well.

### 3.2. Comparison of Pre- and Post-Intervention Status

#### 3.2.1. Emotion Regulation

To examine if a change in ER skills occurred after the intervention, the CERQ and DERS subscale scores were entered as dependent variables in a repeated-measures analysis of variance (rANOVA) using the Benjamini–Hochberg significance to decrease the false discovery rate due to multiple comparisons [73]. Moreover, the assumption of normality was met. Regarding the total score of DERS, participants showed a statistically significant improvement in ER skills, F(1, 19) = 6.77, *p* = 0.018, and ηp^2^ = 0.27. They showed statistically significant changes in several specific ER skills, including increased access to emotion regulation strategies (confidence in that they can find multiple ways to manage negative emotions), F(1, 18) = 11.17, *p* = 0.004, and ηp^2^ = 0.38, and decreased self-blame, F(1, 17) = 12.71, *p* = 0.002, and ηp^2^ = 0.43 (see Table 2). The changes in other skills did not reach significance, but an examination of the means shows a trend towards improvement post-intervention. These included reduced catastrophizing (Mpre = 10.89, Mpost = 9.53, *p* = 0.09), an increased ability of emotional acceptance (Mpre = 14, Mpost = 11.56, *p* = 0.029), increased concentration and accomplishment of tasks (Mpre = 16.06, Mpost = 14.11, *p* = 0.08), and reduced impulse control difficulties, (Mpre = 18.47, Mpost = 16.11, *p* = 0.09).

#### 3.2.2. Symptom Reduction

The results from a one-way rANOVA using the Benjamini–Hochberg significance showed that there was a significant effect from pre- to post-intervention on the participants’ scores for internalizing and externalizing problems, F(1, 22) = 5.98, *p* = 0.023, and ηp^2^ = 0.21; and F(1, 22) = 10.75, *p* = 0.003, and ηp^2^ = 0.33, respectively. More specifically, we found a significant reduction in affective problems (Mpre = 9.17, Mpost = 7.09, *p* = 0.002), reported ADHD symptoms (Mpre = 6, Mpost = 5.04, *p* = 0.02), and conduct problems (Mpre = 8.87, Mpost = 7.04, *p* = 0.012). In contrast, the effect of time did not reach significance on participants’ scores for oppositional defiant problems (Mpre = 5.04, Mpost = 4.43, *p* = 0.04), anxiety, (Mpre = 3.74, Mpost = 3.35, *p* = 0.26), and somatic problems (Mpre = 2.65, Mpost = 2.65, *p* = 1.0) (see Table 2).

With regard to substance use, 13 participants reported that they smoked before the intervention and 14 stated that they used alcohol regularly. Participants’ scores for nicotine dependence were in the upper level of moderate dependence before the intervention. The rANOVA, with the total Fagerstrom score as the dependent variable, showed that, overall, participants showed a significant decrease in scores, *p* = 0.002, (Mpre = 5.23, Mpost = 4.23). After the intervention, nicotine dependence dropped to the lowest level of moderate dependence. Significant effects were also observed in the reduction in alcohol dependence, as measured by the total AUDIT scores, *p* = 0.012 (Mpre = 5.21, Mpost = 2.71, Table 2).

## 4. Discussion

A feasibility pilot study was carried out in a naturalistic school setting having as a main goal the enhancement of ER skills in adolescents. A short-term group intervention was designed for adolescents at risk of psychopathology as assessed by their schools, based on ER-focused elements of different CBT approaches. The direct involvement of school counselors ensured that the intervention was introduced to students who might benefit the most, and that it would be delivered with the support of the school, which may have been critical in securing consent to participate as well as the low dropout.

We found that adolescents overall described the treatment as useful and participated in almost all sessions with minimal dropout, while counselors reported satisfaction with the intervention. Specifically, adolescents found the intervention very useful, enjoyable, and a unique opportunity to learn about emotions and their regulation. Most of them noted that the intervention during school hours helped them to participate and express their thoughts, emotions, and experiences, which, in other cases, they could not. The naturalistic setting apparently helped to increase adolescents’ motivation for participation as shown by the low dropout, high completion of the program, satisfaction, and acceptability [74,75]. Only one participant dropped out from the intervention after the first session showing the importance of the naturalistic setting. In addition, the intervention was rated as very useful and to be recommended to schoolmates.

In terms of outcomes, adolescents showed improved ER skills and reduced emotional, behavioral, and substance use problems after the program. The results suggest that this is a promising intervention for adolescents at risk for mental health problems or addiction. The intervention’s novelty lies in the combination of core ER skills derived from different CBT perspectives and in taking a transdiagnostic approach, providing them with a wide repertoire of skills to allow for flexible coping [21].

The findings support that participants demonstrated improvement in several specific ER skills after the program, such as confidence in finding multiple ways to manage in the face of difficult emotions (i.e., increased coping flexibility) and reduced self-blame. Naturally, given the absence of a control group in the context of this preliminary feasibility study, the results cannot be etiologically attributed to the intervention. However, as the changes were consistent with the aims, i.e., showing an increase in adaptive ER skills and decreases in maladaptive coping (e.g., self-blame), it appears that the program had an impact on ER. This remains to be verified through future RCTs but the results are encouraging [76,77]. The fact that this study did not find significant changes in other ER subscales, such as impulsivity, rumination, putting into perspective, catastrophizing, and other-blame, requires some further consideration. This can be explained perhaps by the fact that other skills were emphasized more in this prevention program, or to the small sample size of this preliminary application that did not allow effects to reach significance, despite a trend in the expected direction.

Preliminary findings are also encouraging with regard to the decrease in various symptoms, though future RCTs, with follow-up assessments over several months or years, will provide more definitive answers. Notably, externalizing problems were found to improve more than internalizing problems. Potential reasons for this are that participants had more externalizing problems than internalizing problems in this sample, allowing more room for an effect. Additionally, externalizing problems frequently co-occur with internalizing problems, such as anxiety and depression (e.g., [57,78,79]), so that the improvement in internalizing symptoms found in previous studies may, in part, be due to the changes in externalizing problems. Furthermore, the intervention targeted skills relevant to interpersonal situations and anger that may be better at reducing externalizing problems.

The current study must be seen in light of an important limitation, which is the small sample and the absence of a no-treatment or treatment-as-usual control group, which is, however, an inherent limitation of early-stage feasibility research. The inclusion of a wait-list or treatment-as-usual control group and the recruitment of more participants in a future formal RCT will allow for the more definitive attribution of obtained improvements to the intervention rather than to maturation or other causes. However, given the findings of improved ER skills, a known mechanism of psychopathology, it is likely that, indeed, the improvement in the symptoms came about from better ER and a wider coping repertoire. Another limitation for the continuation of the program is that the counsellors will need to be trained to implement it and may find it difficult to include it in the curriculum due to time constrains; students who participate in the program may need to extend their normal school hours.

## 5. Conclusions

In sum, this study represents an early-stage application of a short-term, ER-focused intervention for adolescents at risk, aiming to examine its feasibility, acceptability, and effectiveness estimation. It was found to be feasible within the school setting and acceptable to adolescents. Future plans will include conducting RCTs to formally examine the efficacy of the intervention, with the intention of developing a usable psychosocial program that can be applied in the school context, where access may be easier, dropout rates lower, and where school counselors can determine the suitability of the program for specific students facing psychosocial risk. The program’s preliminary results are promising and stress the importance of removing obstacles to attendance by bringing interventions close to adolescents’ naturalistic settings, so that uptake and engagement are improved.

## Figures and Tables

**Table 1:** Content of the emotion regulation intervention sessions.

Session	Topic	Session Components	ER Skill
1	Introduction	Ice-breaking and getting to know each other Training objectives and group rules Setting personal goals	
2	Understanding emotions	Explaining the cognitive behavioral model (Thought–Emotion–Behavior) The need for emotion regulation	Emotion recognition
3	Acceptance of emotions	Myths about emotions Recognizing emotions without attempts to change them. Exercise: Observing emotions without attempts to change them	Acceptance skills
4	Thinking styles	Recognizing unhelpful thinking styles Seeing a situation from different perspectives Exercise: Practice alternative interpretations of a situation and observe feelings change	Cognitive restructuring
5	Sadness and anxiety management	Recognize feelings of sadness and anxiety Changing emotions by doing the opposite Exercise: Relaxation techniques	Behavioral activation
6	Anger management and problem solving	What is anger? Recognizing emotions that come before feeling angry Exercise: Skills to communicate anger and find solutions	DBT skills

**Table 2:** Changes from pre- to post-intervention in emotion regulation and mental health problems.

	*F*	*p*	*η_p_* ^2^
DERS			
Non-acceptance of emotional responses	5.67	0.03	0.25
Difficulties engaging in goal directed behavior	3.45	0.08	0.17
Impulse control difficulties	3.22	0.09	0.15
Lack of emotional awareness	0.31	0.58	0.02
Limited access to emotion regulation strategies	11.17	0.01	0.38
Lack of emotional clarity	2.25	0.15	0.12
CERQ			
Self-blame	12.27	0.01	0.42
Acceptance	2.15	0.160	0.11
Rumination	2.82	0.11	0.14
Positive refocusing	0.00	0.95	0.01
Refocus on planning	0.83	0.37	0.05
Positive reappraisal	0.92	0.35	0.05
Putting into perspective	1.56	0.23	0.08
Catastrophizing	3.19	0.09	0.15
Other-blame	1.17	0.29	0.06
YSR problems			
Affective problems	12.97	0.01	0.37
Anxiety problems	1.30	0.26	0.06
Somatic problems	0.00	1.00	0.01
ADHD	6.17	0.02	0.22
Oppositional defiant problems	4.52	0.05	0.17
Conduct problems	7.56	0.01	0.26
Nicotine dependence	15.6	0.01	0.57
Alcohol dependence	8.52	0.01	0.40

## Data Availability

Data is unavailable due to privacy.
